# “Prognostic Significance of Neutrophil‐to‐Lymphocyte, Platelet‐to‐Lymphocyte, and Monocyte‐to‐Lymphocyte Ratios in Myeloid Neoplasms: A Systematic Review”

**DOI:** 10.1002/hsr2.73049

**Published:** 2026-08-26

**Authors:** Mahsa Dabir, Ali Abdolbaghaei, Fatemeh Zoghi, Fatemeh Karpasand, Ali Maleki

**Affiliations:** ^1^ Student Research Committee, Kermanshah University of Medical Sciences Kermanshah Iran; ^2^ Student Research Committee, Faculty of Nursing and Midwifery Lorestan University of Medical Sciences Khorramabad Iran; ^3^ School of Nursing and Midwifery Kermanshah University of Medical Sciences Kermanshah Iran; ^4^ Department of Medical Laboratory Science School of Paramedical, Kermanshah University of Medical Sciences Kermanshah Iran

**Keywords:** acute myeloid leukemia, monocyte‐to‐lymphocyte ratio, myelodysplastic syndromes, neutrophil‐to‐lymphocyte ratio, platelet‐to‐lymphocyte ratio, prognostic biomarkers, systematic review

## Abstract

**Background and Aims:**

Myelodysplastic syndromes (MDS) and acute myeloid leukemia (AML) are heterogeneous hematologic malignancies with variable outcomes. Inflammatory markers from routine blood counts, including neutrophil‐to‐lymphocyte ratio (NLR), platelet‐to‐lymphocyte ratio (PLR), and monocyte‐to‐lymphocyte ratio (MLR), have emerged as potential prognostic biomarkers. This systematic review aimed to evaluate their prognostic and diagnostic significance in MDS and AML.

**Methods:**

A systematic search of PubMed, Web of Science, and Scopus was performed from inception to June 2026. Studies evaluating NLR, PLR, or MLR in MDS or AML were included. Lymphocyte‐to‐monocyte ratio values were converted to MLR using MLR = 1/LMR. Data were synthesized narratively due to heterogeneity.

**Results:**

Thirteen studies (2847 patients) met inclusion criteria. Elevated NLR, PLR, and MLR were consistently associated with shorter overall survival. NLR showed independent prognostic value in AML and higher‐risk MDS (HR: 1.05–2.136). MLR demonstrated the most consistent prognostic association (cutoffs: 0.20–0.67). PLR showed context‐dependent value (cutoffs: 22.14–213.2). Only one study assessed diagnostic performance, reporting excellent discrimination for NLR (AUC = 0.9169) and good performance for PLR (AUC = 0.8312).

**Conclusion:**

NLR, PLR, and MLR are accessible biomarkers that may complement risk stratification in MDS and AML. However, heterogeneity in cutoff values limits routine clinical use. Prospective standardized studies are needed to validate these findings.

## 1 Introduction

Myelodysplastic syndromes (MDS) and acute myeloid leukemia (AML) are closely related hematologic disorders. MDS is characterized by ineffective hematopoiesis, leading to peripheral cytopenias such as anemia, neutropenia, and thrombocytopenia [1]. A significant proportion of MDS patients, particularly those with higher‐risk disease, can progress to AML, with progression rates ranging from 20% to 40% depending on the risk category [2, 3]. The transformation to AML is marked by an increase in blast cells in the bone marrow to ≥ 20% [1]. Globally, the incidence of AML has risen significantly, increasing from 79,372 cases in 1990 to 144,645 cases in 2021 [4], accounting for approximately 23.1% of all leukemia cases worldwide [5].

Prognosis in AML and MDS is highly variable, influenced by genetic abnormalities, disease burden, and patient‐related factors [6, 7]. Risk stratification systems such as the International Prognostic Scoring System (IPSS), the WHO Prognostic Scoring System, the Revised IPSS (IPSS‐R), and the Molecular IPSS (IPSS‐M) have been developed to categorize patients based on cytogenetics, blast percentage, and molecular mutations to guide treatment decisions [8]. However, despite advances in these models, predicting disease trajectory remains challenging, particularly for patients with intermediate‐risk disease. Moreover, the implementation of IPSS‐M, despite its refined prognostic capabilities, is limited by the high cost and technical requirements associated with next‐generation sequencing, making IPSS‐R the most widely utilized scoring system in clinical practice [9, 10].

Recent evidence suggests that chronic inflammation and immune dysregulation play a pivotal role in the pathogenesis and progression of MDS and AML [11, 12]. Inflammatory cytokines contribute to hematopoietic suppression, clonal evolution, and leukemic transformation, highlighting the importance of immune‐mediated mechanisms in disease progression [13]. Given this, systemic inflammatory markers have gained attention as potential prognostic indicators in hematologic malignancies [14]. Among these, hematologic indices derived from routine blood tests, such as the neutrophil‐to‐lymphocyte ratio (NLR), platelet‐to‐lymphocyte ratio (PLR), monocyte‐to‐lymphocyte ratio (MLR), and lymphocyte‐to‐monocyte ratio (LMR), have been explored as surrogate markers of inflammation and immune status in malignancies [15].

NLR, in particular, has been studied extensively in AML. Zhang et al. [16] reported that an elevated NLR serves as an independent predictor of poor treatment response in non‐M3 AML patients with a high percentage of bone marrow blast. Similarly, lymphopenia, represented by a low LMR, has been associated with adverse clinical outcomes across different cancers [17]. Despite their accessibility and cost‐effectiveness, the prognostic significance of these markers in MDS and AML remains controversial, with conflicting findings across studies [18, 19]. Furthermore, these indices may be influenced by factors such as infections, corticosteroid use, and concurrent inflammatory conditions, potentially confounding their clinical interpretation [20]. This systematic review aims to comprehensively evaluate the prognostic significance of NLR, PLR, MLR, and LMR in patients with MDS and AML. By synthesizing current evidence, we seek to determine whether these hematologic indices can enhance prognostic stratification beyond existing scoring systems and provide clinically relevant insights for individualized risk assessment and treatment decision‐making. Additionally, we will explore the potential limitations and confounding factors affecting these markers to ensure an evidence‐based and clinically applicable interpretation of their prognostic value.

## 2 Methods

### 2.1 Study Design and Registration

**Figure 1:**
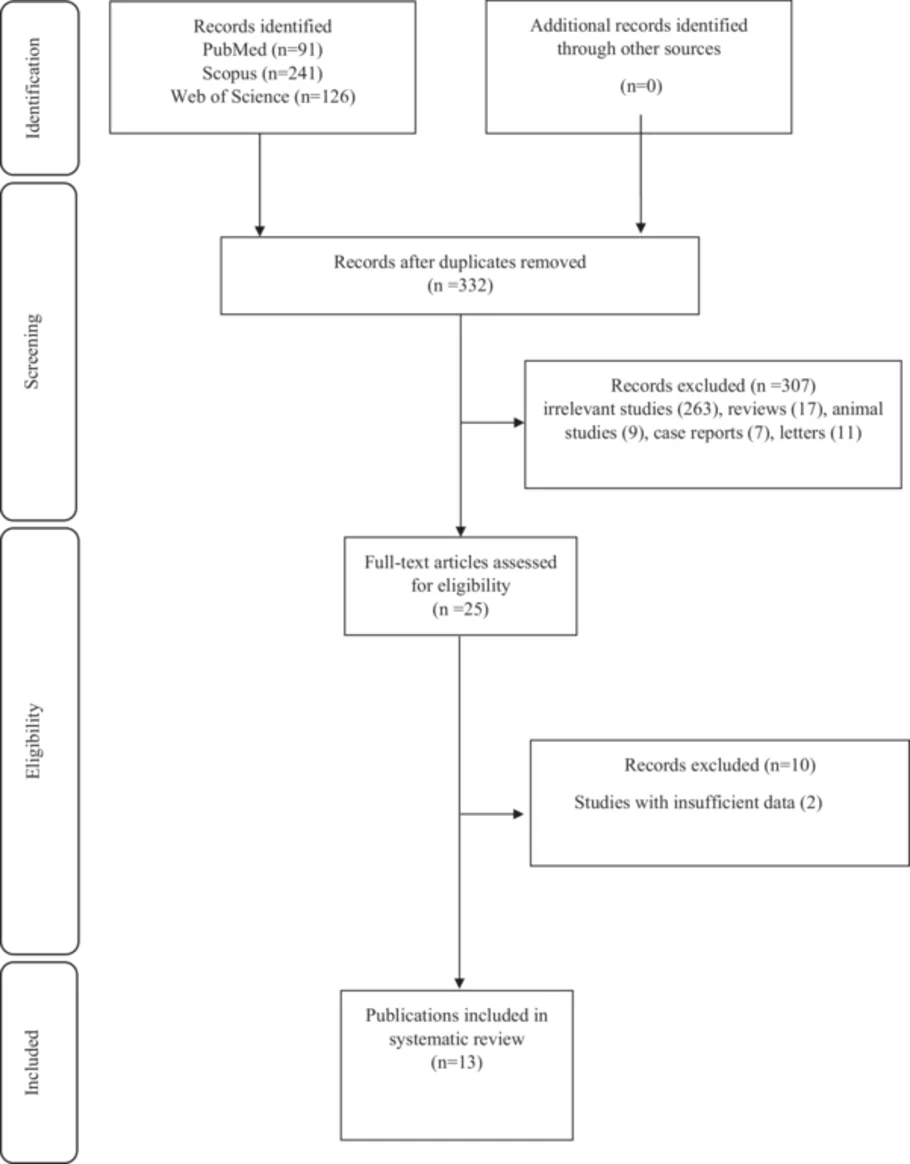
Flow diagram of the literature search and selection process according to the PRISMA guidelines.

This systematic review was conducted in accordance with the Preferred Reporting Items for Systematic Reviews and Meta‐Analyses (PRISMA) guidelines (Figure 1). As the study was based exclusively on previously published data and did not involve direct human participation, ethical approval and informed consent were not required.

### 2.2 Search Strategy

A comprehensive literature search was performed in PubMed, Web of Science, and Scopus to identify relevant studies published from database inception to June 2026. The search strategy combined terms related to inflammatory biomarkers and myeloid malignancies. The inflammatory marker search terms included “Neutrophil‐to‐Lymphocyte Ratio”, “Neutrophil‐Lymphocyte Ratio”, “neutrophil lymphocyte ratio”, “NLR”, “PLR”, “platelet‐to‐lymphocyte ratio”, “platelet lymphocyte ratio”, “LMR”, “lymphocyte‐to‐monocyte ratio”, “lymphocyte to monocyte ratio”, “MLR”, “monocyte‐to‐lymphocyte ratio”, and “monocyte to lymphocyte ratio”. The disease‐related search terms included “MDS”, “myelodysplastic syndrome”, “myelodysplastic syndromes”, “AML”, “acute myeloid leukemia”, and “acute myeloid leukaemia”. The complete search strategy for each database is provided in Supporting Information S1: Table S1.

### 2.3 Eligibility Criteria

Studies were considered eligible if they met all of the following criteria: original research articles published in peer‐reviewed journals; evaluation of the prognostic or diagnostic value of NLR, PLR, LMR, or MLR in patients with MDS or AML; and reporting of relevant clinical outcomes, including survival, treatment response, or diagnostic performance. Studies were excluded if they met any of the following criteria: duplicate publications; case reports, review articles, letters, editorials, commentaries, or expert opinions; experimental, laboratory‐based, or animal studies; conference abstracts, meeting reports, or proceedings lacking peer review; unavailable full‐text articles; insufficient data for extraction; or publications in languages other than English.

### 2.4 Study Selection and Data Extraction

Two reviewers independently screened titles and abstracts for eligibility. Full texts of potentially relevant articles were subsequently assessed for inclusion. Any discrepancies were resolved through discussion and consensus, with consultation from a third reviewer when necessary. Data extracted from each eligible study included first author, publication year, country, study design, disease type, sample size, median age, sex distribution, follow‐up duration, evaluated biomarkers, biomarker cutoff values, cutoff determination methods, study endpoints, and effect estimates. Effect measures included hazard ratios, odds ratios, 95% confidence intervals, and corresponding *p* values. Additional information collected included the significance of biomarkers in univariate and multivariate analyses, variables used for adjustment, and diagnostic performance measures such as area under the receiver operating characteristic curve, sensitivity, and specificity when available.

### 2.5 Standardization of Biomarker Reporting

To ensure consistency in the direction of biomarker effects across studies, all reported LMR values were converted to MLR using the formula MLR = 1/LMR. This conversion was performed because elevated MLR values consistently reflect a more pronounced inflammatory state, characterized by relatively higher monocyte counts or lower lymphocyte counts. Consequently, higher MLR values correspond to poorer prognostic profiles, facilitating direct comparison with NLR and PLR, for which increased values are similarly associated with adverse outcomes. Studies originally reporting either LMR or MLR were therefore presented uniformly as MLR throughout the data tables and narrative synthesis.

### 2.6 Quality Assessment and Risk of Bias

The methodological quality and risk of bias of included studies were assessed using the Quality In Prognosis Studies tool. This instrument evaluates six domains: study participation, study attrition, prognostic factor measurement, outcome measurement, study confounding, and statistical analysis and reporting. Each domain was rated as having a low, moderate, or high risk of bias. Overall study quality was determined based on the collective pattern of ratings across all domains.

### 2.7 Data Synthesis

Given the substantial heterogeneity among studies with respect to patient populations, disease subtypes, biomarker cutoff values, outcome definitions, and statistical methodologies, quantitative meta‐analysis was considered inappropriate and was therefore not performed. Instead, findings were synthesized narratively and summarized in structured tables. Primary outcomes of interest included overall survival (OS), progression‐free survival, leukemia‐free survival, complete remission, treatment response, and diagnostic performance metrics such as area under the curve, sensitivity, and specificity. Results were organized according to biomarker type and disease category.

### 2.8 Statistical Analysis

As this is a systematic review without primary data collection or statistical modeling, no formal statistical analysis was performed on aggregate data. All reported effect estimates (hazard ratios, odds ratios, and 95% confidence intervals) were extracted directly from the original studies and presented as reported by the authors. No new statistical tests or meta‐analytic pooling were conducted. Therefore, no statistical software was used for data analysis. The *p* values reported in this review are those provided by the original studies and were not recalculated. For consistency with journal guidelines, all *p* values are presented according to standard conventions (*p* < 0.001; *p* values between 0.001 and 0.01 to the nearest thousandth; *p* values ≥ 0.01 to the nearest hundredth; and *p* > 0.99 as reported).

## 3 Results

### 3.1 Study Selection and Characteristics

The study selection process is presented in Figure 1. A total of 13 full‐text original studies met the eligibility criteria and were included in this systematic review. The characteristics of the included studies are summarized in Table 1. The studies were published between 2014 and 2026. Twelve studies employed a retrospective cohort design [14, 16, 21, 22, 23, 24, 25, 26, 27, 28, 29, 30], whereas one study was prospective [31].

**Table 1:** Characteristics of included studies.

Study	Year	Country	Study design	Disease	Sample size (*n*)	Median age (years)	Follow‐up
Akinci et al.	2014	Turkey	Retrospective	MDS	40	70	NR
Saeed et al.	2017	USA	Retrospective	Primary MDS	889	72	27 months
Zahran et al.	2020	Egypt	Prospective	Pediatric Acute Leukemia	73	9	NR
Yikilmaz et al.	2020	Turkey	Retrospective	MDS	62	68.5	62.8 ± 4.5 months
Zhang et al.	2021	China	Retrospective	De novo non‐M3 AML	181	40	28 months
Pénichoux et al.	2021	France	Retrospective	Primary MDS	201	75	27 months
Shi et al.	2022	China	Retrospective	MDS	213	62	NR
Lu et al.	2023	China	Retrospective	MDS	91	65	NR
Qu et al.	2024	China	Retrospective	Primary MDS	334	59	NR
Hu et al.	2025	China	Retrospective	MDS	70	59	2 years
Cai et al.	2025	China	Retrospective	MDS	110	72	NR
Stefaniuk et al.	2025	Poland	Retrospective	De novo AML	204	54.5*	NR
Lee et al.	2026	Taiwan	Retrospective	Primary MDS	554	67.3	NR

Abbreviations: AML, acute myeloid leukemia; MDS, myelodysplastic syndromes; NR, not reported.

Sample sizes ranged from 40 to 889 patients (median = 140). Six studies included patients with MDS [14, 21, 22, 23, 24, 29], four focused on primary MDS [25, 26, 27, 30], two investigated de novo non‐M3 AML [16, 28], and one evaluated pediatric acute leukemia [31]. Median patient age ranged from 9 to 75 years. Follow‐up duration was reported in four studies [22, 23, 25, 27], with median follow‐up ranging from 27 to 62.8 months, whereas the remaining studies provided incomplete follow‐up information.

### 3.2 Risk of Bias Assessment

The results of the risk of bias assessment using the QUIPS tool are presented in Table 2. Overall, most studies were judged to have a low risk of bias across the domains of study participation, study attrition, prognostic factor measurement, outcome measurement, and statistical analysis.

**Table 2:** Risk of bias assessment of included studies using the QUIPS tool.

Study (first author, year)	Study participation	Study attrition	PF measurement	Outcome measurement	Study confounding	Statistical analysis	Overall RoB
Akinci et al., 2014	Moderate	Low	Low	Low	High	Low	High
Cai et al., 2025	Low	Low	Low	Low	Low	Low	Low
Hu et al., 2025	Low	Low	Low	Low	Low	Low	Low
Lee et al., 2026	Low	Low	Low	Low	Low	Low	Low
Lu et al., 2023	Low	Low	Low	Low	Low	Low	Low
Pénichoux et al., 2021	Low	Low	Low	Low	Low	Low	Low
Qu et al., 2024	Low	Low	Low	Low	Low	Low	Low
Saeed et al., 2017	Low	Low	Low	Low	Low	Low	Low
Shi et al., 2022	Low	Low	Low	Low	Low	Low	Low
Stefaniuk et al., 2025	Low	Low	Low	Low	Low	Low	Low
Yikilmaz et al., 2020	Moderate	Low	Low	Low	High	Low	High
Zahran et al., 2020	Moderate	Low	Moderate	Low	Low	Moderate	Moderate
Zhang et al., 2021	Low	Low	Low	Low	Low	Low	Low

Two studies [21, 22] were rated as having a high risk of bias because of inadequate adjustment for confounding factors. One study [31] was considered to have a moderate risk of bias due to limitations in prognostic factor measurement and statistical analysis. The remaining 10 studies were rated as low risk of bias across all assessed domains.

### 3.3 Neutrophil‐to‐Lymphocyte Ratio

Four studies evaluated the prognostic significance of NLR (Table 3A). Zhang et al. [16] identified NLR ≥ 2.0 as an independent adverse prognostic factor for both OS and event‐free survival (EFS) in patients with de novo non‐M3 AML. In addition, the adverse prognostic effect of elevated NLR remained consistent across different induction chemotherapy regimens, including IA, DA, and HA protocols [16]. Zahran et al. [31] reported an association between higher NLR values and lower complete remission rates in pediatric acute leukemia; however, survival outcomes were not evaluated. In contrast, Akinci et al. [21] found no significant association between NLR (cutoff = 1.8) and mortality among patients with MDS (*p* = 0.75). Reported NLR cutoff values ranged from 1.8 to 2.0 [31]. Stefaniuk et al. [28] demonstrated that higher NLR was independently associated with no response to induction chemotherapy in patients with de novo AML (OR = 1.05, 95% CI: 1.02–1.09, *p* = 0.006), but the association with OS was not significant in multivariate analysis. Reported NLR cutoff values ranged from 1.8 to 4.222.

**Table 3A:** Prognostic significance of inflammatory biomarkers in MDS and AML.

Study	Biomarker	Cutoff	Outcome	Effect estimate	Significant association
Akinci et al. 2014	NLR	1.8	Overall survival	Not reported	No (*p* = 0.75)
Saeed et al. 2017	MLR	≤ 0.20	Overall survival	HR: 1.20 (95% CI: 1.02–1.40)	Yes (univariate only)*
Zahran et al. 2020	NLR	Not reported	Complete remission	Higher NLR → lower remission rates	Yes
Yikilmaz et al. 2020	PLR	46	Overall survival	Not reported	No (*p* = 0.596)
Zhang et al. 2021	NLR	2.0	OS and EFS	Independent adverse prognostic factor	Yes
Pénichoux et al. 2021	MLR	0.20–0.33	Leukemia‑free survival	HR: 1.03 (95% CI: 1.01–1.05)	Yes
Shi et al. 2022	PLR	22.14	Overall survival	HR: 1.898 (95% CI: 1.059–3.399)	Yes
Lu et al. 2023	MLR	> 0.31	Overall survival	HR: 1.872 (95% CI: 1.084–3.230)	Yes
Qu et al. 2024	PLR	213.2	Overall survival	HR: 2.136 (95% CI: 1.154–3.953)	Yes
Cai et al. 2025	MLR	> 0.125	Overall survival	*p* = 0.011 (multivariable)	Yes
Stefaniuk et al. 2025	MLR	Continuous (median ~1.01)	OS/Response	Univariate OS—HR: 0.94 (*p* = 0.010)	No (multivariable)
Stefaniuk et al. 2025	NLR	Continuous	Response (NR)	OR: 1.05 (95% CI: 1.02–1.09)	Yes
Lee et al. 2026	MLR	< 0.67**	OS and LFS	OS—HR: 1.484 (*p* = 0.014)	Yes (OS)

*Note:* LMR values were converted to MLR using MLR = 1/LMR for standardization. Higher MLR indicates a more inflammatory state. *Statistical significance was observed only in the univariate analysis. **Cutoff originally reported as LMR and converted to MLR using MLR = 1/LMR.

* Statistical significance was observed only in the univariate analysis.

** Cutoff originally reported as LMR and converted to MLR using MLR = 1/LMR.

**Table 3B:** Diagnostic and exploratory findings [23].

Marker	Cutoff	AUC	*p* value
NLR	4.222	0.9169	< 0.0001
PLR	19.58	0.8312	0.0004
MLR	NR	0.6286	0.1723 (NS)

Abbreviation: NS = not significant.

**Table 4:** Evidence synthesis of inflammatory biomarkers across included studies.

Biomarker	Number of studies	Main findings	Reported prognostic cutoffs
NLR	6 (1 independent OS, 1 response, 4 non‐independent/negative)	Zhang (2021) independent OS in AML subgroup; Stefaniuk (2025) independent for response (NR) in AML; Shi (2022) univariate only; Zahran (2020) CR association; Hu (2025) diagnostic only; Akinci (2014) negative	1.8–4.222
PLR	4 (2 positive, 2 negative)	Shi (2022) and Qu (2024) independent OS; Yikilmaz (2020) negative; Stefaniuk (2025) univariate OS only (KM)	22.14–213.2
MLR	6 (4 independent OS, 1 univariate only, 1 diagnostic)	Lu (2023), Cai (2025), Lee (2026) independent OS; Pénichoux (2021) independent LFS; Saeed (2017) univariate only; Hu (2025) diagnostic only	0.20–0.67

### 3.4 Platelet‐to‐Lymphocyte Ratio

Four studies assessed the prognostic value of PLR (Table 3A). Shi et al. [14] reported that PLR > 22.14 was associated with shorter OS (HR = 1.898, 95% CI: 1.059–3.399). Similarly, Qu et al. [26] demonstrated that PLR ≥ 213.2 independently predicted worse OS (HR = 2.136, 95% CI: 1.154–3.953). However, Yikilmaz et al. [22] observed no significant association between PLR (cutoff = 46) and OS in patients with MDS (*p* = 0.6) [22]. Stefaniuk et al. [28] reported that lower PLR was associated with shorter OS in Kaplan–Meier analysis (*p* = 0.006), but this association did not remain significant in multivariate analysis [28]. Reported PLR cutoff values ranged from 22.14 to 213.2.

### 3.5 Monocyte‐to‐Lymphocyte Ratio

Six studies evaluated MLR (Tables 3A and 4). Saeed et al. [27] reported that MLR ≤ 0.20 was associated with shorter OS in univariate analysis (HR = 1.20, 95% CI: 1.02–1.40); however, the association was no longer significant after adjustment for the Revised International Prognostic Scoring System (IPSS‐R). Pénichoux et al. [25] found that higher MLR values independently predicted inferior leukemia‐free survival (HR = 1.03 per 0.01 increase, 95% CI: 1.01–1.05). Likewise, Lu et al. [24] demonstrated that MLR > 0.31 independently predicted worse OS in patients with MDS (HR = 1.872, 95% CI: 1.084–3.230). Cai et al. [29] reported that MLR > 0.125 was independently associated with worse OS in MDS (*p* = 0.01). Lee et al. [30] demonstrated that an elevated L/M ratio (MLR < 0.67) independently predicted worse OS (HR = 1.484, *p* = 0.01) after adjustment for IPSS‐R, IPSS‐M, and WHO‐2022/ICC classifications. Stefaniuk et al. [28] reported that higher MLR was associated with shorter OS in univariate analysis (HR = 0.94, 95% CI: 0.89–0.98, *p* = 0.01) but did not remain significant in multivariate analysis. Reported cutoff values ranged from 0.20 to 0.67 (Table 3A).

### 3.6 Diagnostic Performance of Inflammatory Biomarkers

Only one study [23] evaluated the diagnostic performance of inflammatory biomarkers (Table 3B). NLR demonstrated excellent discriminatory ability for differentiating patients with MDS from non‐MDS cytopenic controls (cutoff = 4.222; AUC = 0.9169, *p* < 0.001). PLR also showed good diagnostic performance (cutoff = 19.58; AUC = 0.8312, *p* < 0.001). In contrast, MLR did not demonstrate statistically significant diagnostic utility (AUC = 0.6286, *p* = 0.17) [23].

### 3.7 Findings According to Treatment Setting

Evidence regarding treatment‑specific effects was limited (Table 3A). Zhang et al. [16] reported that the adverse prognostic impact of elevated NLR was consistent across different induction chemotherapy regimens, including IA (Idarubicin + Cytarabine), DA (Daunorubicin + Cytarabine), and HA (Homoharringtonine + Cytarabine) protocols. Lee et al. [30] demonstrated that the adverse prognostic effect of an elevated L/M ratio (MLR < 0.67) was mitigated by allogeneic hematopoietic stem cell transplantation (HSCT) but not by hypomethylating agents (HMAs). No study directly compared biomarker performance between intensive chemotherapy and HMA‑based therapies. An overall synthesis of the prognostic evidence for NLR, PLR, and MLR across the included studies is presented in Table 4.

## 4 Discussion

This systematic review synthesized the prognostic and diagnostic significance of three routine inflammatory biomarkers—NLR, PLR, and MLR—in patients with MDS and AML. After standardizing LMR to MLR using the formula MLR = 1/LMR, data from 13 full‐text studies were analyzed. The findings consistently demonstrate that elevated inflammatory ratios are associated with adverse clinical outcomes, although the strength and independence of these associations vary across biomarkers, reflecting differences in disease biology, patient selection, treatment exposure, and methodological approaches across studies.

**Figure 2:**
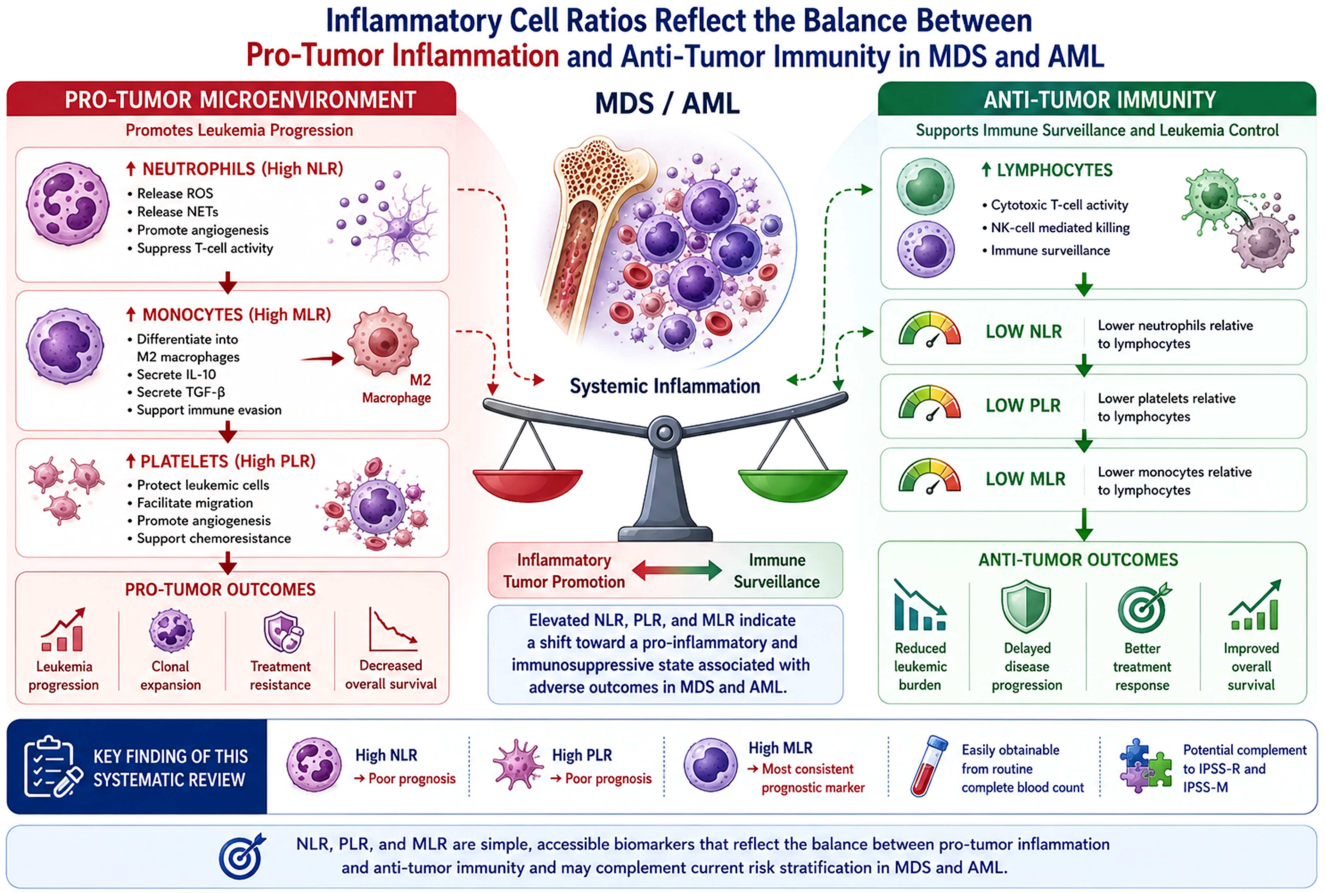
Central Illustration. Balance between Pro‐Tumor Inflammation and Anti‐Tumor Immunity in MDS and AML. Elevated NLR, PLR, and MLR indicate a shift toward a pro‐inflammatory and immunosuppressive state associated with leukemia progression, treatment resistance, and inferior survival, whereas lymphocyte‐mediated immune surveillance contributes to disease control and improved outcomes.

The prognostic associations observed in the present review are biologically plausible and consistent with current understanding of the immune microenvironment in myeloid malignancies. Neutrophils may promote leukemic progression through the release of inflammatory mediators, angiogenic factors, and immunosuppressive molecules. Platelets can support malignant cell survival and facilitate interactions between leukemic cells and the bone marrow microenvironment [32, 33]. In contrast, lymphocytes play a central role in antitumor immune surveillance; therefore, a relative reduction in lymphocyte counts may reflect impaired immune control of disease [34]. Similarly, increased monocyte counts may indicate enhanced differentiation toward tumor‐supportive macrophage populations [35]. Consequently, elevated NLR, PLR, and MLR may represent a systemic inflammatory state associated with disease progression and inferior clinical outcomes in patients with MDS and AML. The proposed biological mechanisms linking inflammatory cell ratios to disease progression are summarized in the Central Illustration (Figure 2).

Among the three markers, MLR demonstrated the most consistent prognostic association, with multiple independent cohorts confirming its value even after adjustment for established risk models such as IPSS‐R and IPSS‐M. This consistency suggests that MLR captures clinically meaningful alterations in immune‐inflammatory balance that are not fully accounted for by traditional scoring systems. The biological basis is supported by transcriptomic evidence showing downregulation of inflammatory pathways (IL‐2–STAT5, IL6–JAK–STAT3) and p53 signaling in patients with elevated MLR, providing mechanistic insights into the adverse prognosis observed in this group [30]. Lee et al. [30] further demonstrated that the adverse effect of elevated MLR was mitigated by allogeneic HSCT but not by HMAs, suggesting that inflammatory markers may predict response to specific therapeutic strategies [29]. A large meta‐analysis of 63 studies across cancer types confirmed that high MLR was significantly associated with shorter progression‐free survival (HR: 1.50) and OS (HR: 1.52), supporting the robustness of this marker across different malignancies [36].

NLR, despite being the most extensively studied, showed the greatest variability; positive associations were primarily observed in AML and higher‐risk MDS, while studies in lower‐risk or heterogenous MDS populations often failed to demonstrate independent significance. This heterogeneity likely reflects differences in disease biology and patient selection rather than absence of a true biological effect, as broader meta‐analytic evidence—including an umbrella review across multiple cancer types—confirmed that elevated NLR is consistently associated with poor OS and cancer‐specific survival [36, 37]. A large multicentre analysis of 4347 patients with MDS further demonstrated that NLR ≥ 4 independently predicted inferior OS after adjustment for IPSS‐M and was associated with lower response rates to HMAs, underscoring the clinical relevance of this marker in higher‐risk populations [38]. PLR occupied an intermediate position, with positive findings largely confined to MDS cohorts and inconsistent results in AML. A meta‐analysis of 244 studies across various cancer types demonstrated that elevated PLR is closely associated with poor OS [37]. however, evidence in haematological malignancies remains less robust, and a meta‐analysis in multiple myeloma found insufficient evidence for PLR as an independent biomarker, possibly because platelet counts are more susceptible to reactive changes from comorbidities, infections, and treatment‐related effects that may dilute their prognostic specificity [39].

Regarding diagnostic utility, only one study evaluated the performance of these markers, reporting excellent discrimination for NLR and good performance for PLR in distinguishing MDS from non‐MDS cytopenic controls, while MLR showed no diagnostic value [23]. Although these findings are promising, the limited evidence currently available and the absence of external validation preclude firm conclusions regarding their diagnostic utility in clinical practice. Evidence regarding treatment‐specific effects was limited. The adverse prognostic impact of elevated NLR appeared consistent across different induction chemotherapy regimens [16], while MLR's prognostic value was mitigated by allogeneic HSCT but not by HMAs [30]. No study has directly compared biomarker performance across contemporary treatment modalities such as venetoclax‐based or oral hypomethylating regimens, representing an important knowledge gap given the rapidly evolving therapeutic landscape of MDS and AML.

An important observation arising from this review is the substantial heterogeneity in reported biomarker thresholds. For prognostic studies, NLR cutoffs ranged from 1.8 to 4.222, PLR cutoffs from 22.14 to 213.2, and MLR cutoffs from 0.20 to 0.67. This variability reflects differences in patient populations, disease subtypes, laboratory methodologies, statistical approaches, and methods used to define optimal thresholds. Despite this heterogeneity, the consistency of directional associations across independent cohorts—particularly for MLR—provides a strong rationale for further prospective validation. Future studies should aim to standardize cutoff determination methods, integrate these markers into existing prognostic scores such as IPSS‐R and IPSS‐M, and prospectively evaluate their predictive value for response to novel therapies.

Several limitations must be acknowledged. First, only 13 studies met the inclusion criteria, limiting the overall evidence base. Second, most included studies employed retrospective designs, increasing susceptibility to selection bias and residual confounding. Third, considerable heterogeneity existed regarding patient populations, disease subtypes, outcome definitions, and biomarker thresholds, precluding formal meta‐analysis. In addition, molecular data were not uniformly incorporated across studies, and adjustment for established prognostic scoring systems varied. Fourth, diagnostic performance was evaluated in only a single study without external validation. Fifth, treatment heterogeneity was not uniformly accounted for, and no study specifically addressed the impact of HMAs or venetoclax. Despite these limitations, the consistency of directional associations across independent cohorts supports the biological relevance and potential clinical value of these biomarkers.

## 5 Conclusion

Elevated baseline NLR, PLR, and MLR are generally associated with poorer survival in patients with MDS and AML, with MLR demonstrating the most consistent prognostic value. In prognostic studies, NLR cutoffs ranged from 1.8 to 4.222, PLR cutoffs from 22.14 to 213.2, and MLR cutoffs from 0.20 to 0.67. These inexpensive and widely available biomarkers may complement existing risk models, particularly in resource‐limited settings where molecular diagnostics are unavailable. However, heterogeneity in study design and biomarker thresholds currently limits routine clinical implementation. Prospective validation in homogeneous cohorts and within the context of modern therapies is required before clinical application can be recommended.

## Author Contributions

M.D. and A.M. designed the study. M.D. and A.A. conducted the literature search and data extraction. F.Z., M.D., and F.K. wrote the original draft. A.M. critically reviewed and edited the manuscript. All authors have read and approved the final manuscript.

## Funding

The authors have nothing to report.

## Disclosure

All authors have read and approved the final version of the manuscript. The corresponding author had full access to all of the data in this study and takes complete responsibility for the integrity of the data and the accuracy of the data analysis.

## Ethics Statement

Ethical approval was not applicable for this study as it is a systematic review of previously published data.

## Consent

The authors have nothing to report.

## Conflicts of Interest

The authors declare no conflicts of interest.

## Transparency Statement

The corresponding author affirms that this manuscript is an honest, accurate, and transparent account of the study being reported; that no important aspects of the study have been omitted; and that any discrepancies from the study as planned have been explained.

## Supporting information


Supporting File

## Data Availability

The data used to support the findings of this study are available from the corresponding author upon reasonable request.
