# Supplementary material for: “Prognostic Significance of Neutrophil‐to‐Lymphocyte, Platelet‐to‐Lymphocyte, and Monocyte‐to‐Lymphocyte Ratios in Myeloid Neoplasms: A Systematic Review”
Source: Health Sci Rep. 2026 Aug 26;9(9):e73049. doi: 10.1002/hsr2.73049 (PMC13519096; doi:10.1002/hsr2.73049)
Supplement: Supplementary file 1 [file HSR2-9-e73049-s001.docx]

**Supplementary** **Table 1**. Complete search strategy with database-specific terms.

| terms | |
| --- | --- |
| ("Neutrophil-to-Lymphocyte Ratio" OR "Neutrophil-Lymphocyte Ratio" OR "neutrophil lymphocyte ratio" OR "NLR" OR " PLR" OR "platelet-to-lymphocyte ratio" OR "platelet lymphocyte ratio" OR "LMR" OR "lymphocyte-to-monocyte ratio" OR "lymphocyte to monocyte ratio" OR "MLR" OR "monocyte-to-lymphocyte ratio" OR "monocyte to lymphocyte ratio") | ("MDS" OR "myelodysplastic syndrome" OR "myelodysplastic syndromes" OR "AML" OR "acute myeloid leukemia" OR "acute myeloid leukaemia" |

March, 2025

| PubMed | #1 AND #2 | 91 |
| --- | --- | --- |
| Scopus | #1 AND #2 | 241 |
| Web of Science | #1 AND #2 | 126 |
| All |  | 458 |
| Duplicates |  | 126 |
